# Targeting ER stress/PKA/GSK-3β/β-catenin pathway as a potential novel strategy for hepatitis C virus-infected patients

**DOI:** 10.1186/s12964-023-01081-9

**Published:** 2023-05-08

**Authors:** Dong Lin, Yijia Chen, Ali Riza Koksal, Srikanta Dash, Yucel Aydin

**Affiliations:** 1 Department of Pathology and Laboratory Medicine, Tulane University School of Medicine, New Orleans, LA 70112 USA; 2 The College of Liberal Arts and Sciences, Arizona State University, Tempe, AZ 85281 USA

**Keywords:** Chronic hepatitis C virus (HCV) infection, Hepatocellular carcinoma (HCC), Direct-acting antiviral agents (DAA), Wnt/β-catenin, Endoplasmic reticulum stress (ER stress), Protein kinase A (PKA), Glycogen synthase kinase-3β (GSK-3β)

## Abstract

**Background:**

Chronic hepatitis C virus (HCV) infection causes hepatocellular carcinoma (HCC). The HCC risk, while decreased compared with active HCV infection, persists in HCV-cured patients by direct-acting antiviral agents (DAA). We previously demonstrated that Wnt/β-catenin signaling remained activated after DAA-mediated HCV eradication. Developing therapeutic strategies to both eradicate HCV and reverse Wnt/β-catenin signaling is needed.

**Methods:**

Cell-based HCV long term infection was established. Chronically HCV infected cells were treated with DAA, protein kinase A (PKA) inhibitor H89 and endoplasmic reticulum (ER) stress inhibitor tauroursodeoxycholic acid (TUDCA). Western blotting analysis and fluorescence microscopy were performed to determine HCV levels and component levels involved in ER stress/PKA/glycogen synthase kinase-3β (GSK-3β)/β-catenin pathway. Meanwhile, the effects of H89 and TUDCA were determined on HCV infection.

**Results:**

Both chronic HCV infection and replicon-induced Wnt/β-catenin signaling remained activated after HCV and replicon eradication by DAA. HCV infection activated PKA activity and PKA/GSK-3β-mediated Wnt/β-catenin signaling. Inhibition of PKA with H89 both repressed HCV and replicon replication and reversed PKA/GSK-3β-mediated Wnt/β-catenin signaling in both chronic HCV infection and replicon. Both chronic HCV infection and replicon induced ER stress. Inhibition of ER stress with TUDCA both repressed HCV and replicon replication and reversed ER stress/PKA/GSK-3β-dependent Wnt/β-catenin signaling. Inhibition of either PKA or ER stress both inhibited extracellular HCV infection.

**Conclusion:**

Targeting ER stress/PKA/GSK-3β-dependent Wnt/β-catenin signaling with PKA inhibitor could be a novel therapeutic strategy for HCV-infected patients to overcomes the issue of remaining activated Wnt/β-catenin signaling by DAA treatment.

**Video Abstract**

**Supplementary Information:**

The online version contains supplementary material available at 10.1186/s12964-023-01081-9.

## Background

Hepatitis C virus (HCV) is a major cause of chronic liver disease. Chronic HCV infection has been recognized as a global substantial burden on human health because of the major cause of cirrhosis and hepatocellular carcinoma (HCC) [1, 2]. In recent years, direct-acting antiviral agents (DAA) therapy achieved high rates of sustained virologic response (SVR) up to 95%, which is associated with a greatly reduced risk of mortality and HCC [3, 4]. However, retrospective studies have shown that, despite their efficacy, DAAs are not able to fully eliminate the risk of HCC [5–13]. The appearance of a measurable number of patients who developed HCC with DAA regimen urges the discovery of new antivirals. Viruses are known to make use of the cell’s signaling pathways to facilitated entry, internalization and replication [14]. The cellular factors of these signaling pathways involved in virus infection have been proposed as attractive targets for antiviral agents [15–17]. Among them, kinases are major targets with their inhibitors comprising up to 30% of drug discovery programs in the pharmaceutical industry [18, 19].

Kinase-mediated protein phosphorylation of viral and cellular proteins has been shown to play important roles on viral infection, replication, and cytotoxicity in a host cell [20]. Kinase-mediated protein phosphorylation can regulate a viral protein’s stability, activity, and interactions with other cellular and viral proteins [21]. Upon a viral infection, a number of kinase signaling pathways, such as mitogen-activated protein kinase, Janus kinase/signal transducers and activators of transcription, protein kinase A (PKA) and protein kinase C pathways, are activated by viral proteins [22–26]. Viruses exploit the activated kinase signaling pathways for their own entry, replication and propagation benefits. Studies have indicated the important role of PKA in HCV entry by reorganization of one of the receptors claudin-1 from the plasma membrane to an intracellular vesicular location [27]. HCV infection increased cAMP levels which is required for PKA activation.

The endoplasmic reticulum (ER) is an important processing cellular organelle for proteins that need to undergo posttranslational modification and folding. When protein synthesis is increased, the excess of unfolded proteins in the ER activates an adaptive signal transduction pathway known as unfolded protein response (UPR) to restore ER homeostasis and promote cell survival or to induce apoptosis if ER stress remains unmitigated [28, 29]. In mammalian cells, The UPR comprises three parallel signaling branches: Protein kinase R-like endoplasmic reticulum kinase (PERK), inositol-requiring enzyme 1 (IRE1) and activating transcription factor 6 (ATF6). Once activated, these three branch pathways of the UPR initiate transcriptional and translational signaling to stop protein translation and increase degradation of unfolded proteins and expand the ER membrane to decrease the load of proteins and increase the protein-folding capacity in the ER to promote cell survival. Studies have shown that the UPR has been implicated in a variety of diseases, particularly viral diseases [30]. For example, after entering the host cell, HCV utilizes the ER to complete its life cycles including viral protein synthesis and processing, genome replication and virus assembly [31]. Studies demonstrate that ER stress activates cyclic AMP (cAMP)-dependent protein kinase A (PKA) pathway in mammalian cells and fish [29, 32]. Studies have shown that inhibition of ER stress with inhibitor tauroursodeoxycholic acid (TUDCA) inhibits viral replication, such as influenza A virus [33].

It is well known that Wnt/β-catenin signaling strongly contributes to tumor initiation and progression in a variety of tumor types [34–39]. Under normal physiological conditions, the N-terminus of cytosolic β-catenin is constitutively phosphorylated by glycogen synthase kinase 3 beta (GSK-3β) and casein kinase 1 (CK1) [40]. The phosphorylated β-catenin undergoes ubiquitination and is degraded by the proteasome pathway. Studies demonstrated that GSK-3β activity is inactivated through phosphorylation by protein kinase A (PKA), Akt (also known as protein kinase B), protein kinase C, p70 S6 kinase, and other kinases [41].

In previous studies, we demonstrated chronic HCV infection activated Wnt/β-catenin signaling pathway due to inhibition of GSK-3β activity via serine 9 phosphorylation (p-ser9-GSK-3β) leading to a stable non-phosphorylated β-catenin [42]. We further determined that p-ser9-GSK-3β was PKA-dependent phosphorylation in HCV chronic infection. Unexpectedly, Wnt/β-catenin signaling remained activated in chronic HCV-infected cells after HCV eradication by DAA. Given that DAA could not restore Wnt/β-catenin signaling even though after HCV was eradicated and that Wnt/β-catenin signaling plays an important role in tumorigenesis [34], there is an urgent need to develop therapeutic agents to both eradicate HCV and down-regulate β-catenin levels in HCV-infected patients.

In the present study, we showed that Wnt/β-catenin signaling remained activated after either HCV or HCV replicon eradication by DAA in chronic HCV infection and HCV replicon, respectively. ER stress/PKA/GSK-3β-dependent Wnt/β-catenin pathway existed in both chronic HCV infection and HCV replicon. Inhibition of either PKA activity with PKA inhibitor H89 or ER stress with ER stress inhibitor tauroursodeoxycholic acid (TUDCA) both effectively inhibited HCV replication and reversed Wnt/β-catenin signaling, as well as inhibited extracellular HCV infection. Our results therefore established ER stress/PKA/GSK-3β-dependent Wnt/β-catenin pathway as a novel potential therapeutic target for HCV-infected patients.

## Methods

### Cell culture and HCV infection and drug treatment

Huh-7.5 cells were obtained from the laboratory of Dr. Charles M. Rice (The Rockefeller University, New York). R4-GFP HCV replicon cells were generated in our laboratory [43]. Huh7.5 cell line and R4-GFP HCV replicon cell line were cultured in Dulbecco’s modified Eagle’s medium (DMEM; Life Technologies, Carlsbad, CA) supplemented with 2 mM/L glutamine, sodium pyruvate, nonessential amino acids, 100 U/mL of penicillin, 100 mg/mL of streptomycin, and 10% fetal bovine serum. Huh7.5 cells were infected with JFH-∆V3-EGFP virus (HCV genotype 2a) as previously described [42]. Briefly, Huh7.5 ells were incubated with virus at MOI of 1 for 2 h at 37 °C, washed and incubated with complete DMEM, and these cells were incubated about 6 months and passaged about every 3 days. Cells were collected by day 6(d6), d9, d15, d20, d36, d40, d72, d110, d129, d135, d140, d163, d168 post-infection for either western blotting analysis or drug treatments later. For testing extracellular HCV infection, Huh7.5 cells were infected with infectious extracellular HCV virions in the medium without or with either different doses of H89 (2.5, 5 and10 uM) or different doses of TUDCA (0.5 and 1 mM). Cells were passaged on day 3 post-infection and cultured for additional 4 days with the same dose of either H89 or TUDCA. At the end of treatment, cells were collected for western blotting and cytospin slide preparation. All HCV infection experiments were performed in triplicate. For Akt inhibitor triciribine, mTOR inhibitor rapamycin and PKA inhibitor H89 treatment, chronically HCV infected cells d129, d135 and d140 were treated with different doses of H89 (6, 8, 20, 40 uM), rapamycin (50, 100 and 200 nM) and triciribine (20, 40 and 80 uM), respectively, for 48 h. At the end of drug treatment, cells were collected for western blotting and cytospin slide preparation. For low doses of H89 treatment, chronically HCV infected cells (d168) were treated with low doses of H89 (6 and 8 uM) for 12 days and passaged every 3 days with the same doses of H89. At the end of drug treatment, cells were collected for western blotting. For treatment of R4-GFP HCV replicon cells with H89, cells were treated with 8 uM of H89 for 14 days and 25 days and passaged every 3 days with the same dose of H89. At the end of treatment, cells were collected for western blotting and cytospin slide preparation. For treatment of chronically HCV infected cells and R4-GFP HCV replicon cells with TUDCA, chronically HCV infected cells (d163) and R4-GFP HCV replicon cells were treated with different doses of TUDCA (0.5 and 1 mM) for 48 and 96 h, respectively. At the end of treatment, cells were collected for western blotting.

### Western blotting

At the indicated time points, cells were collected, washed once with PBS and resuspended in RIPA buffer for cell lysate preparation. The whole cell lysates were subjected to electrophoresis on 4–12% Bis–Tris gels. After electrophoresis, proteins were transferred to nitrocellulose membranes, after protein transfer, the resulting nitrocellulose membrane was incubated in blocking buffer, washed in TBST, wrapped with stretch-tite plastic wrap and cut into different strips, each of which covers an area with a protein of interest located in the middle according to the molecular weight of prestained marker on the flanking lanes of the gel, across the entire width of the membrane. The different strips were incubated with different primary antibodies interested including anti-HCV-core (Invitrogen, Waltham, MA, USA, Cat. No. MA−080, diluted 1:1000), β-catenin (Cell Signaling, Danvers, MA, USA, Cat. No. 9562, diluted 1:1000), phospho-GSK-3β (Ser9) (Cell Signaling, Cat. No. 9323, diluted 1:1000), mTOR, phospho-P70 S6 (Cell Signaling, Cat. No. 9205, diluted 1:1000), phosphor-AKT (S473), phospho-PKA substrates (Cell Signaling, Cat. No. 9624, diluted 1:1000), GFP (Santa Cruz, Cat. No. SC-9996, diluted 1:1000), PERK (Cell Signaling, Cat. No. 3192, diluted 1:1000), IRE1α (Cell Signaling, Cat. No. 3294, diluted 1:1000), ATF-6α (Santa Cruz, Cat. No. SC-22799, diluted 1:1000) antibodies, followed by incubation with a secondary antibody, and visutalized by the ECL western blotting detection system (Amersham).

### Cell proliferation assay

For MTT assay, cells were plated at a density of 7500 cells/well in a 96-well plate in 100ul of media without or with H89 or TUDCA treatment and cultured for 48 h. 20 μl of 5 mg/ ml MTT (MTT was prepared in PBS) was added to each well. Cells were incubated for 3.5 h at 37 °C. Remove media and add 150 μl MTT solvent (4 mM HCl, 0.1% of NP-40 all in isopropanol) to the cells. Cover with tinfoil and agitate cells on orbital shaker for 15 min. Read absorbance at 595 nm. For cell counting, seed chronic HCV-infected cells (d168) in growth media about 30% confluence without or with H89 treatment (6 or 8 uM). After reaching 75–80% confluence, they are split 1:3 and grown to 75–80% confluence again without or with the same doses of H89 treatment and repeat this “split-grown” step until day 12. The cells were harvested for cell counting using EVE Automated Cell Counter and western blot.

### Cytospin slide preparation and fluorescence microscopy

Cells were harvested, resuspended in PBS and spun onto cytospin slides in a Cytospin 4 centrifuge (Thermo Scientific) at 1000 rpm for 5 min. The cytospin slides were dried out at room temperature. The cells were mounted using Vecta shield antifade mounting medium with DAPI (VECTOR laboratories). Fluorescence microscopy of green fluorescent protein (GFP)-tagged HCV were obtained by OLYMPUS IX73Microscope System (Tokyo, Japan).

### HCV eradication

Chronic HCV (JFH-∆V3-EGFP)-infected Huh7.5 cells, uninfected control Huh7.5 cells and R4-GFP HCV replicon cells were treated with either a combination of two DAA: Ledipasvir (LED) (400 nM) and Sofosbuvir (SOF) (400 nM) or interferon-α (IFN) (1 IU) to eradicate HCV for three rounds. Cells were passaged every 3 days for each round and cultured with the same doses of drug treatment. After the treatment, cells were collected for western blotting.

### Statistical analysis

Error bars in the figures represent the mean and SD of three biological samples. Student’s t test was performed to evaluate whether the difference between two conditions was significant. Significant differences were marked with ns *P* > 0.05 * *P* ≤ 0.05 ** *P* ≤ 0.01 *** *P* ≤ 0.001 **** *P* ≤ 0.0001.

## Results

### Wnt/β-catenin pathway remains activated after HCV clearance by DAA which triggers Wnt/β-catenin signaling. HCV infection activates PKA/GSK-3β-dependent Wnt/β-catenin pathway

**Fig. 1:**
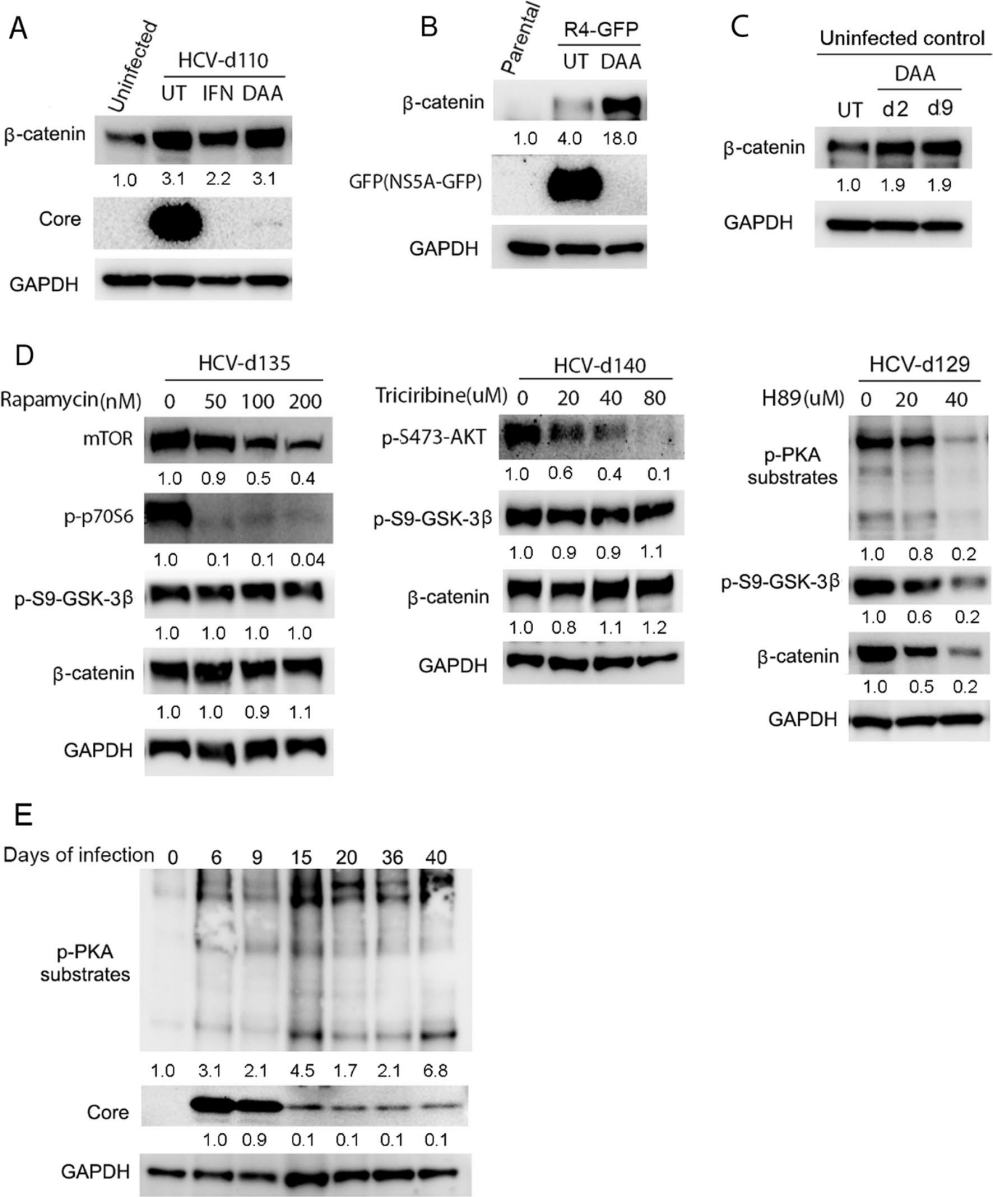
Wnt/β-catenin signaling in chronically HCV-infected Huh7.5 cells and R4-GFP HCV replicon Huh7 cells after HCV clearance by DAA which induced Wnt/β-catenin signaling and chronic HCV infection-induced PKA/GSK-3β/β-catenin signaling in HCV-infected Huh7.5 cells. **A** Chronic HCV-infected Huh7.5 cells (day 110) were treated with either DAA or interferon-α (IFN). After the treatment, cell lysates were collected for western blotting with indicated antibodies. UT, untreated. **B** R4-GFP replicon Huh7 cells were treated with DAA. After the treatment, cell lysates were collected for western blotting with indicated antibodies. Parental, Huh7 cells without HCV replicon. UT, untreated. **C** Uninfected Huh7.5 control cells were treated with DAA. Cell lysates were collected on day 2 and day 9 after treatment for western blotting with indicated antibodies. UT, untreated. **D** Chronic HCV-infected Huh7.5 cells d135, d140 and d129 were treated with mTOR inhibitor rapamycin, AKT inhibitor triciribine and PKA inhibitor H89, respectively. After 48 h, cell lysates were collected for western blotting with indicated antibodies. **E** Huh 7.5 cells were infected with GFP-tagged HCV. Cell lysates were taken at the indicated time points after HCV infection for western blotting with indicated antibodies

In previous studies, we have shown that Wnt/β-catenin signaling is activated in chronic HCV infection [42]. Unexpectedly, β-catenin signaling activation remains even after HCV eradication by direct-acting antiviral agents (DAA) in chronic HCV infection as shown in Fig. 1A. We wanted to test whether this β-catenin signaling activation phenomenon also occurred after HCV clearance by DAA in another cell line. To this end, we used interferon-alpha (INF)-resistant HCV replicon cell line (R4-GFP) [43–45]. These viral self-replicating sub-genomic replicons were derived from sub-genomic viral genomes with deletion of all structural genes. Due to the lack of viral structural genes, viral proteins are not synthesized in the cells resulting no production of infectious viral particles. However, since all trans- and cis-acting elements for HCV viral RNA synthesis are retained, these partial HCV viral RNAs could replicate autonomously in the cells. As shown in Fig. 1B, western blot analysis indicated that Wnt/β-catenin signaling remained activated even after HCV replicon eradication by DAA in R4-GFP HCV replicon cells, consistent with the results obtained from chronic HCV infected cells. Given that Wnt/β-catenin signaling remained activated despite virus or replicon clearance by DAA, one possibility for this phenomenon is that DAA eradicates HCV or replicon and simultaneously activates Wnt/β-catenin signaling. In order to confirm this possibility, we treated the uninfected Huh7.5 control cells with DAA. Western blot analysis indicated that DAA activated Wnt/β-catenin signaling as shown by increased β-catenin levels in uninfected control Huh 7.5 cells (Fig. 1C). Given that β-catenin signaling is a key cancer-associated pathway, development of new antivirals is needed. Our overall goal in this study was to identify novel antiviral drug for HCV that not only eradicates viruses but also inhibits Wnt/β-catenin signaling. Our previous studies showed that the elevated β-catenin protein in chronic HCV infection was due to the inhibition of GSK-3β activity via serine 9 phosphorylation (p-Ser9-GSK-3β) leading to stable non-phosphorylated β-catenin accumulation. Studies indicated that GSK-3β activity is inhibited through phosphorylation of serine 9 by protein kinase A (PKA), Akt (also known as protein kinase B), protein kinase C, p70 S6 kinase, and other kinases [41]. We observed, however, that metformin reversed the β-catenin signaling through PKA/GSK-3β-mediated β-catenin degradation [42]. To further verify the PKA/GSK-3β-mediated β-catenin degradation pathway, three different drugs triciribine (Akt inhibitor), rapamycin (mTOR inhibitor which blocks mTOR-initiated phosphorylation of p70S6 kinase) and H89 (PKA inhibitor) were used to treat chronic HCV-infected cells. Western blotting analysis showed that only H89 PKA inhibitor decreased β-catenin levels as indicated by decreased β-catenin levels in chronic HCV infected-cells (d129) (Fig. 1D), suggesting that p-Ser9-GSK-3β depends on the activation of PKA, instead of AKT and mTOR, leading to activation of Wnt/β-catenin signaling in chronic HCV infection. Given the results above, we hypothesized that HCV infection activates PKA pathway. Western blotting analysis showed that PKA pathway was activated in HCV infection as indicated by increased phosphorylation of PKA substrates (Fig. 1E). Taken together, these results demonstrated that chronic HCV infection activated Wnt/β-catenin signaling in Huh7.5 cells via PKA/GSK-3β-dependent pathway.

### Inhibition of PKA activity with inhibitor H89 both represses HCV replication and reverses Wnt/β-catenin signaling in chronic HCV infection

**Fig. 2:**
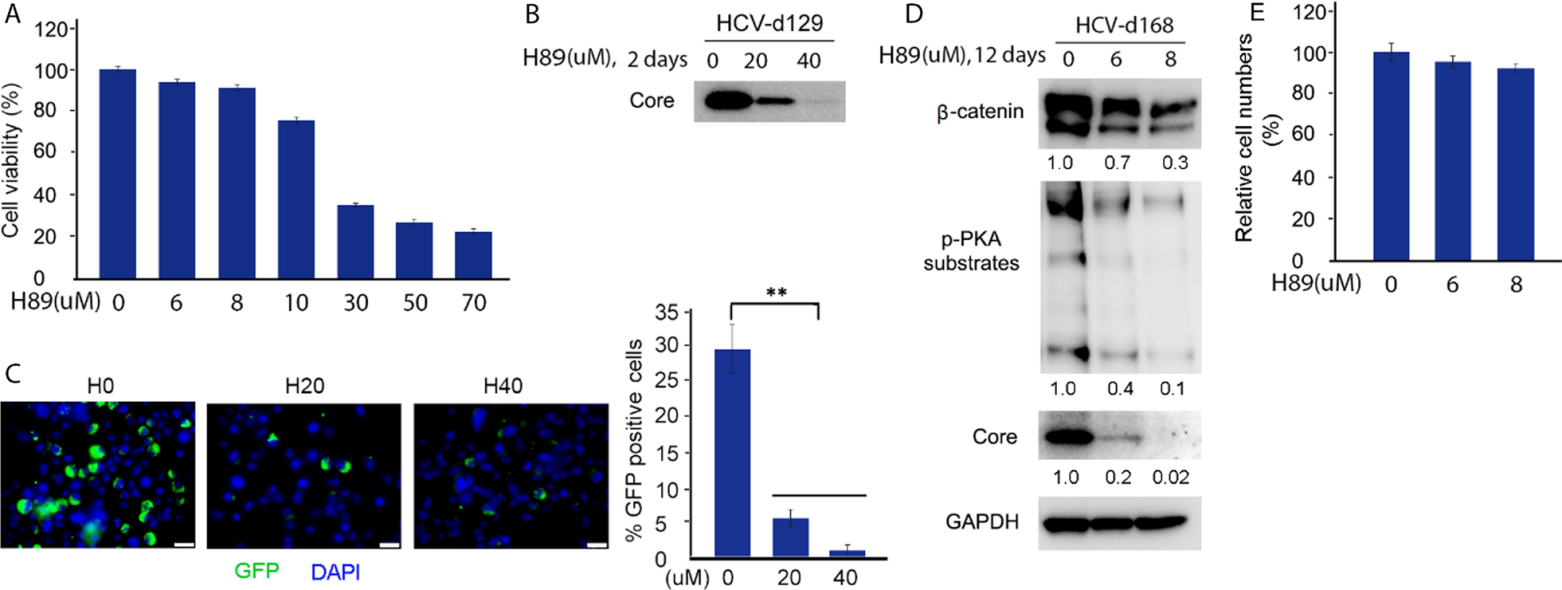
Both repression of HCV replication and reversion of PKA/GSK-3β/β-catenin pathway by PKA inhibition with H89 in chronic HCV-infected Huh7.5 cells. **A** Cytotoxicity of H89 after 48 h of treatment in Chronic HCV-infected Huh7.5 cells (d129) was performed by MTT assay. **B** Chronic HCV-infected Huh7.5 cells (d129) were treated with high doses of H89 (20 and 40 μM). After 48 h, cell lysates were collected for western blotting with indicated antibodies. It is noticed that the internal loading control for western blot analysis are in Fig. 1D. **C** Chronic HCV-infected Huh7.5 cells (d129) were treated with high doses of H89 (20 and 40 μM). After 48 h, fluorescence microscopy was taken. Scale bar = 25 μm. **D** Chronic HCV-infected Huh7.5 cells (d168) were treated with low doses of H89 (6 and 8 μM). After 12 days, cell lysates were collected for western blotting with indicated antibodies. **E** Cell counting assay was performed in chronic HCV-infected Huh7.5 cells treated with low doses of H89 for 12 days

Next, we evaluated whether inhibition of PKA activity both inhibited HCV replication and decreased β-catenin levels. Cytotoxicity of the different concentration of PKA inhibitor H89 in chronic HCV-infected cells was performed. After treatment with H89 at concentrations of 6 and 8 uM, the relative cell viability was above 90%, whereas the relative cell viability was under 80% at the concentrations of more than 10 uM (Fig. 2A). No difference in cell morphology was observed between the treated and untreated cells at a concentration of ≤ 8 uM (data not shown). After 48 h of treatment at high dose of H89 at concentrations of 20 and 40 uM, western blot analysis showed that both the levels of HCV core protein and β-catenin were significantly decreased in dose-dependent manner compared with the untreated control in chronic HCV-infected cells (d129) (Figs. 1D, 2B). Fluorescence microscopy also revealed a marked reduction of HCV-GFP expression in treated cells in a dose-dependent manner compared with the untreated cells (Fig. 2C). For long-term treatment with low dose of H89 at concentrations of 6 and 8 uM, as shown in Fig. 2D, western blot analysis demonstrated that prolonged treatment for 12 days also significantly decreased both the levels of HCV core protein and β-catenin in a dose-dependent manner compared with the untreated control. The concentration of 8 uM completely suppressed viral replication and decreased β-catenin levels in day 12. Although relative cell viability in the cells treated with low dose of H89 at concentrations of 6 and 8 uM for 48 h was above 90%, we still wanted to determine the cytotoxicity after 12-day treatment. We performed cell counting assay for cytotoxicity with the cells treated by low dose of H89 for 12 days. As shown in Fig. 2E, the cell counting assay indicated that the results of the relative cell numbers after 12-day treatment were similar to those of the relative cell viability of H89 treatment for 48 h (6 and 8 uM, cell viability 94 and 91.1%, respectively, for 48 h treatment vs. 95.3 and 92.2%, respectively, for 12-day treatment). These findings reveal a novel therapeutic potential of PKA inhibition with inhibitor H89 for HCV-infected patients to both eradicate HCV and inhibit Wnt/β-catenin pathway activated by chronic HCV infection.

### Inhibition of PKA activity with low dose of inhibitor H89 both inhibits HCV replicon replication and blocks Wnt/β-catenin signaling in R4-GFP HCV replicon cell line

**Fig. 3:**
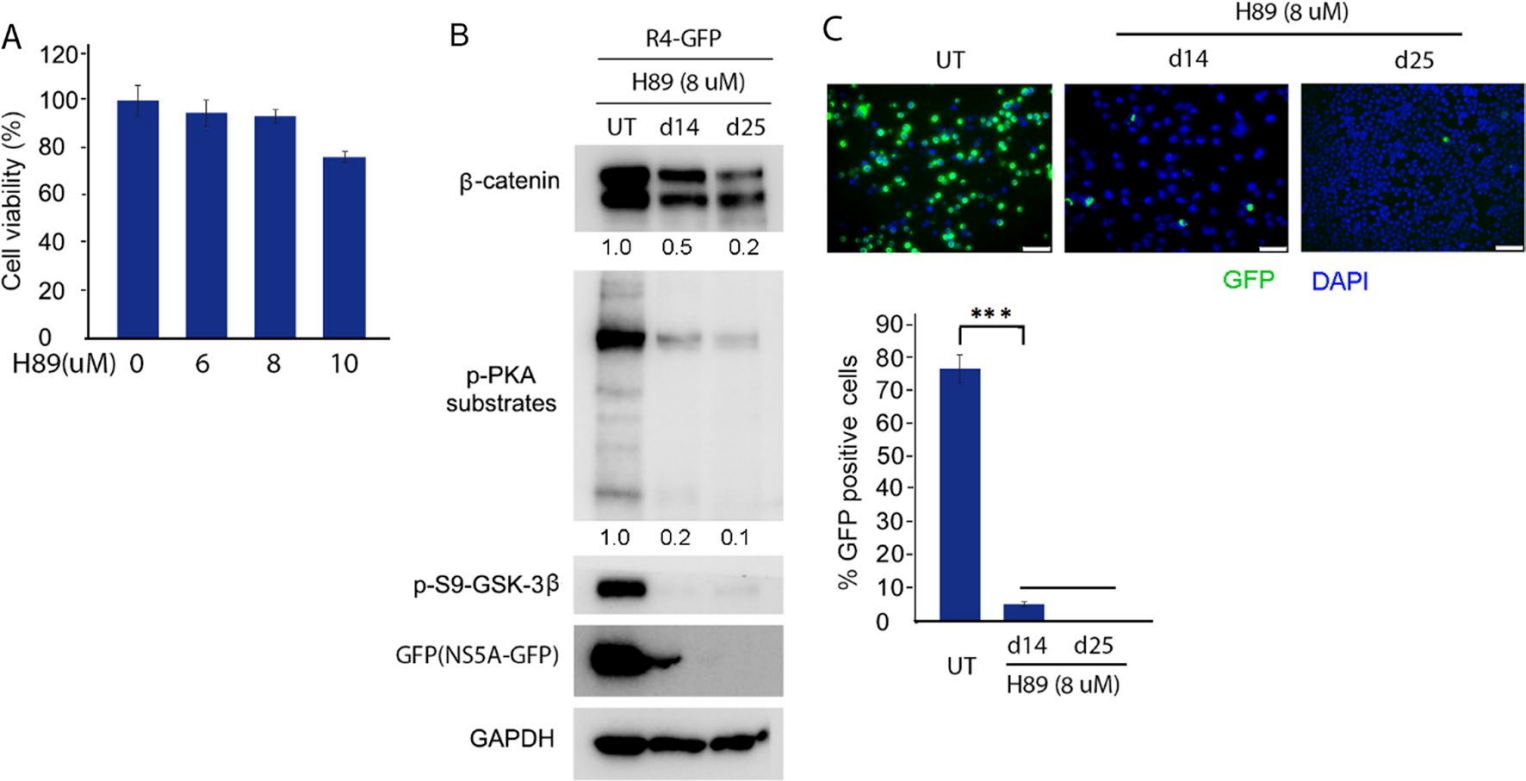
Both repression of HCV replicon replication and reversion of PKA/GSK-3β/β-catenin pathway by PKA inhibition with H89 in R4-GFP HCV replicon Huh7 cells. **A** Cytotoxicity of H89 in R4-GFP HCV replicon Huh7 cells in a concentration-dependent manner by MTT assay. **B** R4-GFP HCV replicon Huh7 cells were treated with low dose of H89 (8 uM). After 14 days and 25 days, cell lysates were collected for western blotting with indicated antibodies. UT, untreated. **C** R4-GFP HCV replicon Huh7 cells were treated with low dose of H89 (8 μM). After 14 days and 25 days, fluorescence microscopy was taken. Scale bar = 50 μm. UT, untreated

Replicon systems have been used in low- and high-throughput screens of compound libraries and have been shown to be reliable to screen and identify viral replication inhibitors [46, 47]. Nonstructural protein 3 (NS3), NS4A, NS4B, NS5A, and NS5B are sufficient for HCV RNA replication in cell culture [48, 49], which is called HCV replicon. NS5A is critical to regulate viral replication and assembly. NS5A is an essential component for the formation of endoplasmic reticulum-derived membranous web where HCV replication takes place. NS5A is known to be highly phosphorylated in cell culture as basally phosphorylated (p56) and hyperphosphorylated (p58) forms [50]. Studies have shown that NS5A phosphorylation is important for HCV replication [51, 52]. The inhibition of NS5A phosphorylation using inhibitors of host cell kinases have been shown to impact HCV replication [53, 54]. Furthermore, the mechanisms of the anti-HCV drug daclatasvir target NS5A by altering the phosphorylation state of the protein [55, 56]. Therefore, inhibition of PKA activity with inhibitor H89 may inhibit HCV replicon replication, as well as block Wnt/β-catenin signaling in R4-GFP HCV replicon cell line as it has in chronic HCV-infected cells. In order to exclude the possibility that the antiviral activity might depend on cytotoxicity of the chemical, a cytotoxicity assay of R4-GFP cells was determined using MTT assay. As shown in Fig. 3A, the results were similar to those achieved in chronic HCV-infected cells with low dose of H89, showing cell viability of > 90% at concentrations of 6 and 8 uM and cell viability of < 80% at concentration of more than 10 uM. The concentrations of H89 without deleterious effects were used for further experiments. H89 treatment at concentration of 8 uM for 14 days markedly both repressed HCV replicon replication as indicated by decreased GFP-tagged HCV NS5A nonstructural protein and inhibited Wnt/β-catenin signaling as shown by reduced β-catenin levels (Fig. 3B). Further repression and inhibition of HCV replication and Wnt/β-catenin signaling, respectively, were observed as the H89 treatment lasted longer up to 25 days (Fig. 3B). Taken together, these results demonstrated that inhibition of PKA activity with low dose of H89 not only repressed HCV replicon replication but also inhibited Wnt/β-catenin signaling in R4-GFP HCV replicon cell line.

### HCV triggers ER stress/UPR pathway, however, inhibition of ER stress by inhibitor TUDCA not only represses HCV but also inhibits ER stress/PKA/GSK-3β-dependent Wnt/β-catenin signaling

**Fig. 4:**
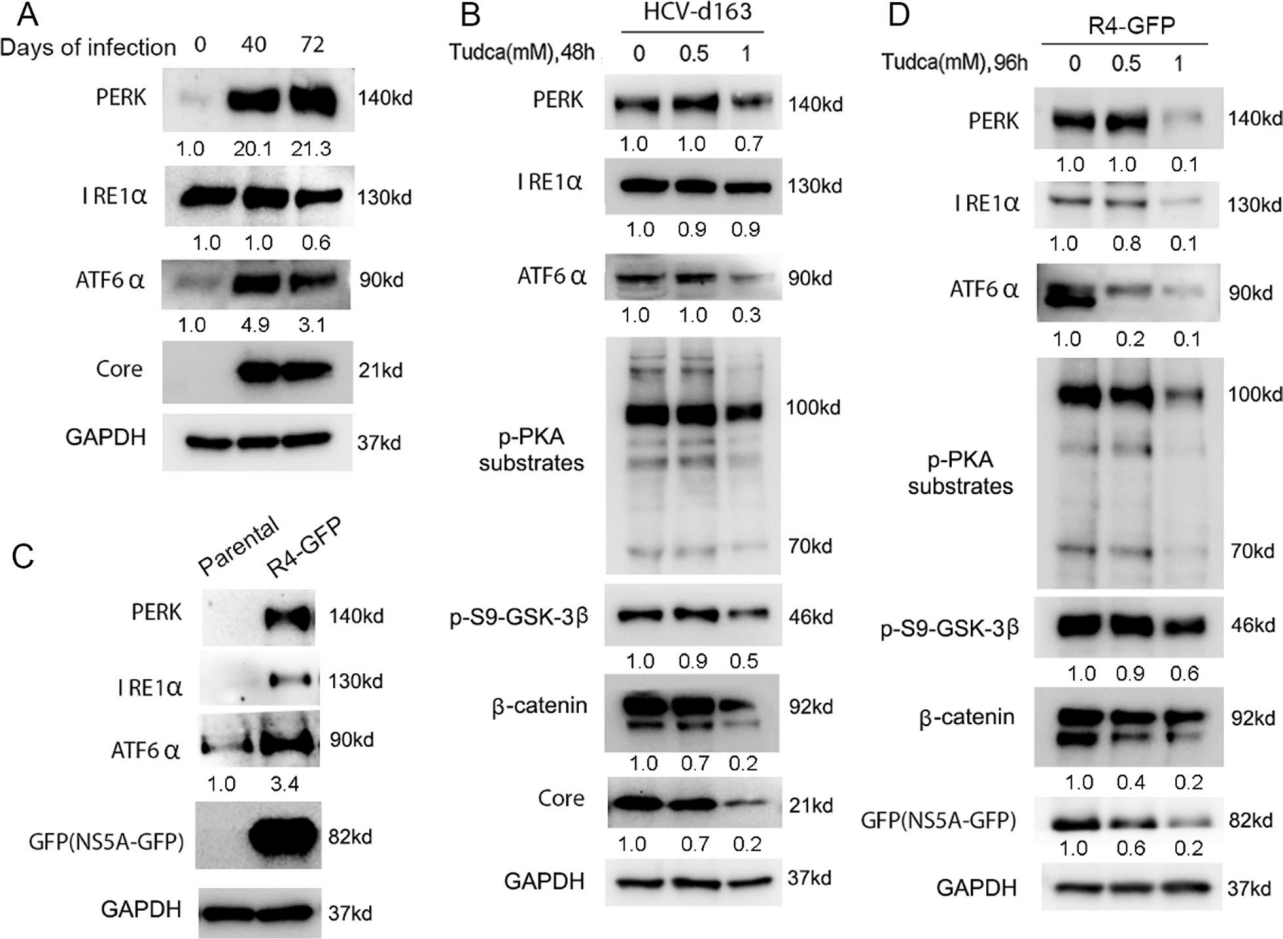
ER stress induction and both repression of HCV replication and reversion of ER stress/PKA/GSK-3β/β-catenin pathway by ER stress inhibitor TUDCA in both chronic HCV-infected Huh7.5 cells and R4-GFP HCV replicon Huh7 cells. **A** Whole cell lysates were taken from chronic HCV-infected Huh7.5 cells (d40 and d72) and uninfected control cells for western blotting with indicated antibodies. **B** Chronic HCV-infected Huh7 cells (d163) were treated with TUDCA. After 48 h, cell lysates were collected for western blotting with indicated antibodies. **C** Whole cell lysates were taken from R4-GFP HCV replicon Huh7 cells and parental cells (without HCV replicon) for western blotting with indicated antibodies. **D** R4-GFP HCV replicon Huh7 cells were treated with TUDCA. After 96 h, cell lysates were collected for western blotting with indicated antibodies

Studies demonstrate that HCV induces formation of a membrane-associated replication complex and HCV proteins associate with endoplasmic reticulum (ER) membranes [57, 58]. HCV replication and assembly in the ER causes ER stress that activates cytoprotective signaling pathway called the unfolded protein response (UPR) mediated by three ER transmembrane proteins PERK, ATF6 and IRE1 [59–61]. However, it is unclear whether PKA/GSK-3β-dependent Wnt/β-catenin signaling is activated by HCV-induced ER stress in chronic HCV infection. To this end, first of all, we investigated if chronic HCV infection triggers ER stress/UPR pathway. As shown in Fig. 4A, western blot analysis revealed that PERK and ATF6α, but not IRE1α, were activated on ER stress in chronic HCV infection as indicated by the upregulation of PERK and ATF6α levels, suggesting that chronic HCV infection triggers ER stress/UPR pathway. In order to eliminate the possibility that ER stress activation by chronic HCV infection is due to the long-term cell culture, we tested the ER stress status with the long-term culture of uninfected Huh7.5 cells.

We showed that there was no significant difference of PERK, ATF6α and IRE1α among do, d40 and d72 (Additional file 1: Figure S1). We next investigated whether inhibition of ER stress affects HCV replication and Wnt/β-catenin signaling in chronic HCV infection. We showed that inhibition of ER stress by TUDCA attenuated both PERK and ATF6α signaling as indicated by decreased both PERK and ATF6α levels, repressed HCV replication as shown by decreased HCV core protein and inhibited PKA/GSK-3β-dependent Wnt/β-catenin signaling as indicated by decreased phosphorylation of both PKA substrates and GSK-3β at serine 9 and β-catenin levels in dose-dependent manner (Fig. 4B), suggesting that chronic HCV infection activated ER stress/PKA/GSK-3β-dependent Wnt/β-catenin pathway, however, inhibition of ER stress with TUDCA not only repressed HCV replication but also inhibited ER stress/PKA/GSK-3β-dependent Wnt/β-catenin pathway. Additionally, MTT assay indicated that TUDCA had low cytotoxicity (Additional file 1: Figure S2). We wanted to know whether this phenomenon also occurred in R4-GFP HCV replicon cells. To this end, we tested whether R4-GFP HCV replicon triggers ER stress. As shown in Fig. 4C, the results of western blot analysis indicated that PERK, ATF6α and IRE1α were robustly activated on ER stress in R4-GFP HCV replicon cells as shown by increased PERK, ATF6α and IRE1α levels, suggesting that HCV replicon triggered ER stress/UPR pathway. Furthermore, we indicated that inhibition of ER stress with TUDCA attenuated ER stress/UPR pathway as indicated by decreased PERK, ATF6α and IRE1α protein levels, inhibited HCV replicon replication as shown by decreased HCV nonstructural protein GFP-tagged NS5A levels and inhibited PKA/GSK-3β-dependent Wnt/β-catenin signaling as shown by decreased phosphorylation of both PKA substrates and GSK-3β at serine 9 and β-catenin levels in dose-dependent manner in R4-GFP HCV replicon cells (Fig. 4D). Taken together, these results demonstrated that either chronic HCV infection or HCV replicon triggered ER stress/UPR pathway, however, inhibition of ER stress by TUDCA not only repressed HCV and HCV replicon replication but also inhibited ER stress/ PKA/GSK-3β-dependent Wnt/β-catenin signaling.

### Targeting ER stress/PKA/GSK-3β-dependent Wnt/β-catenin pathway by inhibition of either PKA activity or ER stress inhibits extracellular HCV infection

**Fig. 5:**
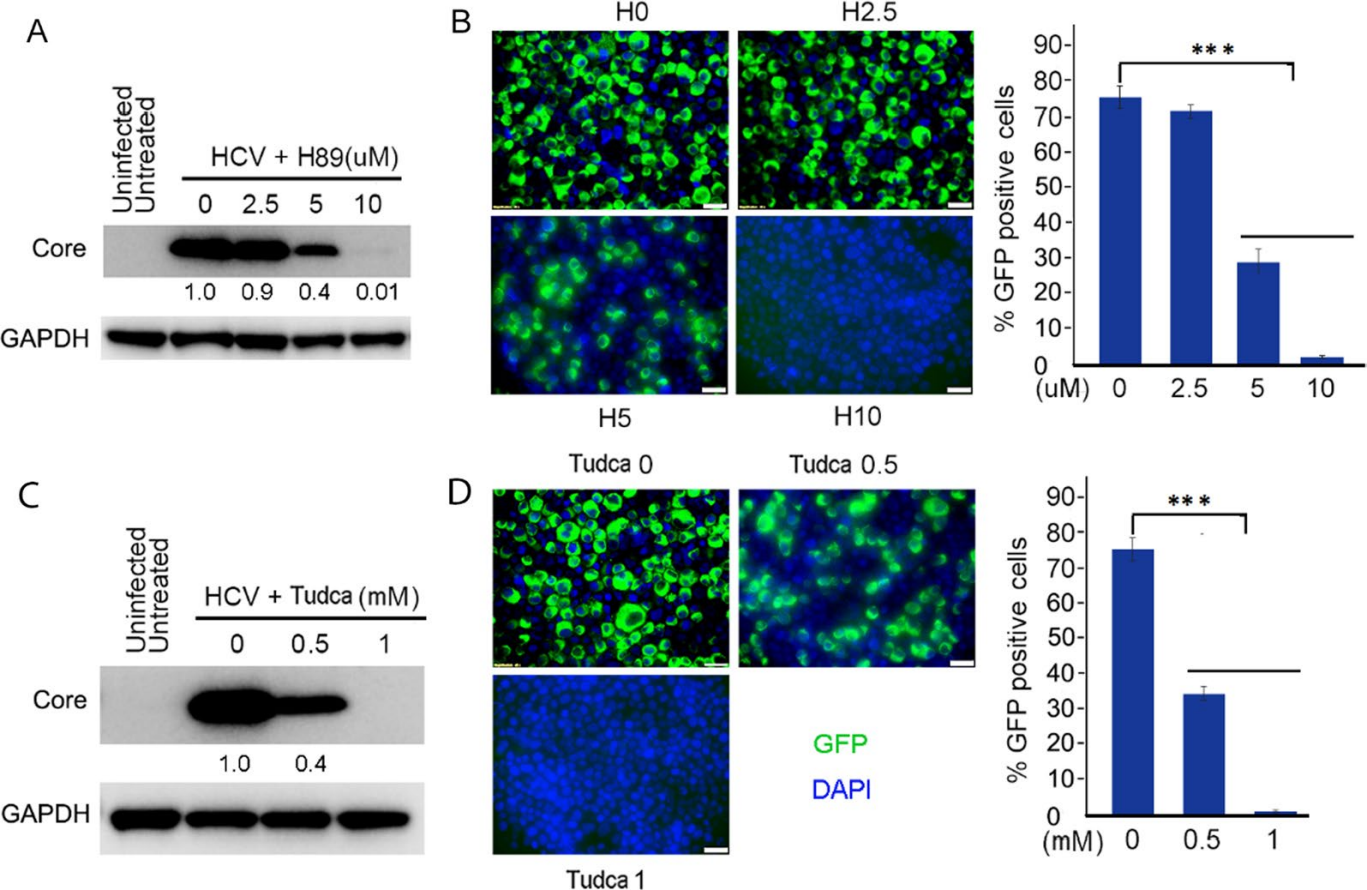
Dose-dependent inhibition of extracellular HCV infection in Huh7.5 cells by targeting ER stress/PKA/GSK-3β/β-catenin pathway by either TUDCA or H89. Huh7.5 cells were infected with contagious extracellular GFP-tagged HCV virions in medium containing different concentration of H89. After 7 days of infection, cell lysates were taken for western blotting with indicated antibodies (**A**) and cells were prepared for cytospin slides, stained with DAPI and carried out imaging microscopy (Scale bar = 25 μm) (**B**). Huh7.5 cells were infected with contagious extracellular GFP-tagged HCV virions in medium containing different concentration of TUDCA. After 7 days of infection, cell lysates were taken for western blotting with indicated antibodies (**C**) and cells were prepared for cytospin slides, stained with DAPI and carried out imaging microscopy (Scale bar = 25 μm.) (**D**)

We next studied the effects of targeting ER stress/PKA/GSK-3β-dependent Wnt/β-catenin signaling with either PKA activity or ER stress inhibitors on inhibition of the infection of infectious extracellular HCV. To investigate the effects of targeting PKA on HCV infection, Huh7.5 cells were infected with infectious extracellular HCV-GFP virions in the presence of different concentrations of PKA inhibitor H89 in the cell culture medium. After 3 days of infection, the medium was changed in the continued presence of PKA inhibitor H89. Cells were incubated for additional 4 days. In the end of 7 days of infection, cells were harvested for both western blot and microscopy of cyto-spin preparation. As shown in Fig. 5A, western blotting analysis indicated dose-dependent reduction of HCV infection by PKA inhibitor H89. Fluorescence microscopy revealed a decrease at concentration of 5 uM and 10 uM H89 by 59.7% (untreated control, 75.1 vs. 5 uM H89 treatment, 30.3, *P* = 0.0009) and 97.3% (untreated control, 75.1 vs. 10 uM H89 treatment, 2.0, *P* = 0.0004), respectively (Fig. 5B), suggesting that PKA inhibitor could effectively inhibit extracellular HCV infection, probably through inhibition of viral either entry or replication or both. Then, we investigated the effects of targeting the ER stress on extracellular HCV infection. As shown in Fig. 5C, western blotting results indicated dose-dependent reduction of extracellular HCV infection by ER stress inhibitor TUDCA (Fig. 5C). Fluorescence microscopy displayed a decrease at concentration of 0.5 mM and 1 mM TUDCA by 52.4% (untreated control, 75.1 vs. 0.5 mM TUDCA treatment, 35.7, *P* = 0.0008) and 98.9% (untreated control, 75.1 vs. 1 mM TUDCA treatment, 0.8, *P* = 0.0003), respectively (Fig. 5D), suggesting that targeting ER stress with inhibitor TUDCA could effectively inhibit extracellular HCV infection, probably through inhibition of viral either entry or replication or both. Taken together, these results demonstrated that targeting the ER stress/PKA/GSK-3β-dependent Wnt/β-catenin pathway with either PKA inhibitor H89 or ER stress inhibitor TUDCA effectively inhibited extracellular HCV infection in Huh7.5 cells.

## Discussion

**Fig. 6:**
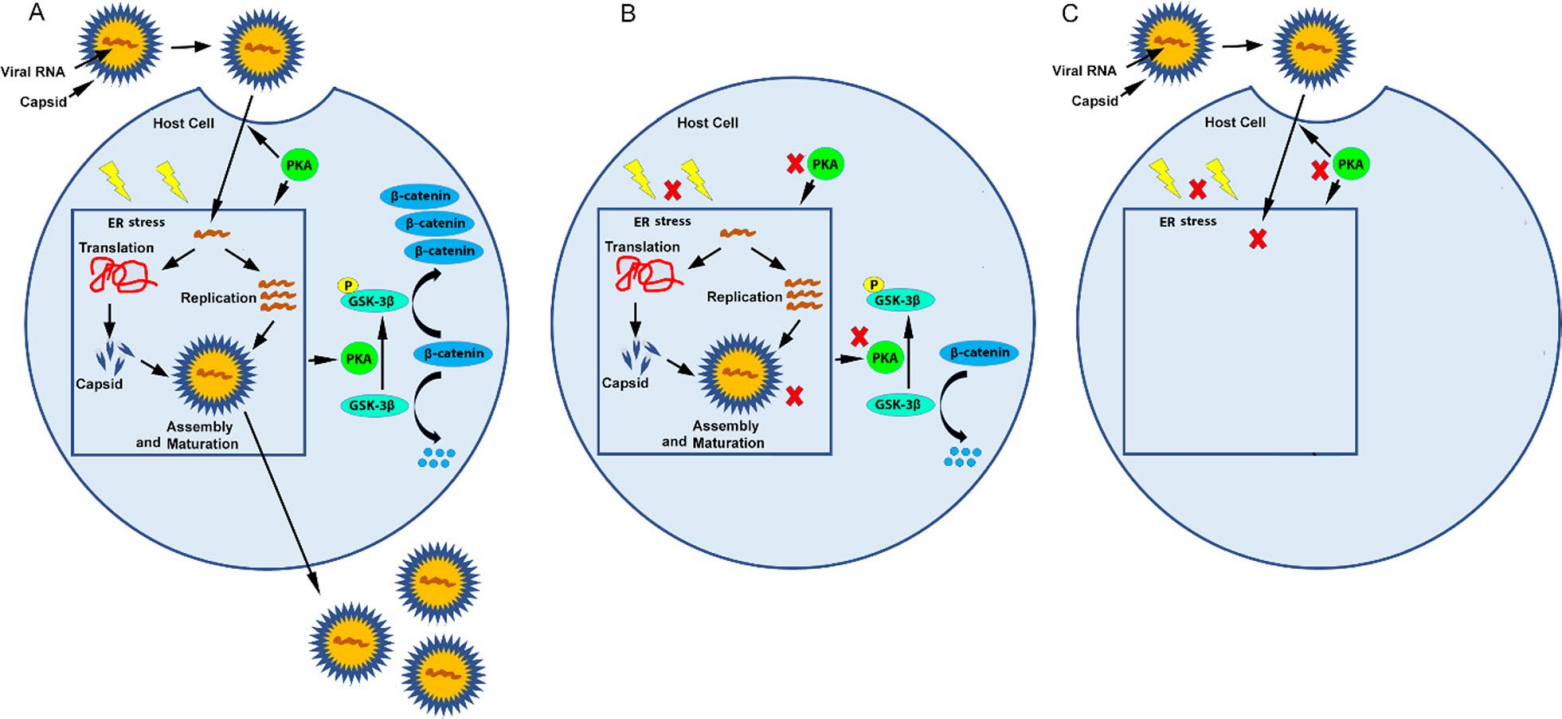
Schematic diagram of the ER stress/PKA/GSK-3β-dependent Wnt/β-catenin pathway. **A** Chronic HCV infection activates ER stress/PKA/GSK-3β-dependent Wnt/β-catenin signaling. **B** Targeting ER stress/PKA/GSK-3β-dependent Wnt/β-catenin pathway by either PKA or ER stress inhibitor both represses HCV replication and reverses Wnt/β-catenin signaling. **C** Targeting ER stress/PKA/GSK-3β-dependent Wnt/β-catenin pathway by either PKA or ER stress inhibitor both inhibits extracellular HCV infection, probably through inhibition of viral either entry or replication or both

Chronic HCV infection has been recognized as the most significant predisposing factors for HCC. DAA is highly effective for HCV treatment. However, retrospective studies have indicated incidence and recurrence of HCC among HCV-associated patients who received DAA treatment despite HCV eradication [5–13, 62, 63]. We previously showed that Wnt/β-catenin signaling is activated in chronic HCV infection [42]. However, Wnt/β-catenin signaling remains activated even after HCV clearance by DAA in chronic HCV-infected cells (Fig. 1A), as well as in R4-GFP HCV replicon cells after HCV replicon clearance by DAA (Fig. 1). Studies have shown that the Wnt/β-catenin signaling strongly contributes to tumor initiation and progression in a variety of tumor types. Thus, there is an urgent need to develop new antiviral strategy for HCV-infected patients to both eradicate HCV and inhibit Wnt/β-catenin signaling. We demonstrated HCV-induced PKA activation and PKA/GSK-3β-mediated, instead of AKT/GSK-3β-mediated and mTOR/GSK-3β-mediated, Wnt/β-catenin signaling in chronic HCV infection (Fig. 1D, E). Inhibition of PKA activity with H89 inhibitor not only repressed HCV replication but also reversed PKA/GSK-3β-mediated Wnt/β-catenin signaling in chronic HCV infection (Fig. 2). As expected, we indicated that inhibition of PKA activity with H89 inhibitor not only repressed HCV replicon replication but also inhibited PKA/GSK-3β-mediated Wnt/β-catenin signaling in R4-GFP HCV replicon cells (Fig. 3). Moreover, we observed that ER stress/UPR pathway was induced by both chronic HCV infection and HCV replicon (Fig. 4A, C). Furthermore, inhibition of ER stress with inhibitor TUDCA not only repressed HCV and HCV replicon replication but also inhibited ER stress/PKA/GSK-3β-dependent Wnt/β-catenin signaling (Fig. 4B, D). Furthermore, targeting the ER stress/PKA/GSK-3β-dependent Wnt/β-catenin pathway with either PKA activity inhibitor H89 or ER stress inhibitor TUDCA effectively inhibited extracellular HCV infection in Huh7.5 cells (Fig. 5). Taken together, these results indicated that chronic HCV infection, as well as R4-GFP HCV replicon, activated ER stress/PKA/GSK-3β-dependent Wnt/β-catenin pathway, and that targeting this pathway with either PKA activity inhibitor H89 or ER stress inhibitor TUDCA not only repressed HCV replication but also reversed the ER stress/PKA/GSK-3β-dependent Wnt/β-catenin signaling in both chronic HCV-infected cells and HCV replicon cells, as well as inhibited extracellular HCV infection (Fig. 6), suggesting that targeting ER stress/PKA/GSK-3β-dependent Wnt/β-catenin pathway may be a potential novel therapeutic strategy for HCV-infected patients.

Studies have indicated that HCV replication is associated with activation of Wnt/β-Catenin Signaling [64]. As a component of the HCV RNA replication complex, NS5A stimulates Wnt/β-catenin pathway directly by binding and stabilizing the β-catenin protein [65], and indirectly by activating the PI3K/Akt signaling, which in turn to mediate the inactivation of GSK3β and stabilization of β-catenin [66–68]. On the other hand, HCV infection promotes microRNA-155 (miR-155) expression, which inhibits APC expression, one of the negative regulators in the destruction complex to regulate the cytoplasmic levels of β-catenin [69].

HCV genome replication occurs at the ER-associated membranous webs [31]. HCV causes massive rearrangements of intracellular membranes in the ER for viral replication and accumulation of unfolded viral proteins [70]. Both studies in vitro and in vivo have indicated that HCV induces ER stress [71–73]. HCV further utilizes ER stress and manipulate the cellular responses to ER stress to promote its persistence and pathogenesis such as HCV-related insulin resistance [74, 75].

In vitro phosphorylation studies have indicated that HCV core protein is a substrate of PKA and protein kinase C [76, 77]. HCV core protein has two phosphorylation sites: serine 53 and serine 116. Phosphorylation of serine 116 by PKA can regulate HCV core levels in infected cells [78]. Farquhar et al. reported that intracellular levels of cAMP, which binds and activates PKA, were increased in HCV-infected cells (27). Studies with viral subgenomic replicons showed that HCV NS2 protein activated cAMP-dependent pathways in Huh7 cells [79]. We and others demonstrated that HCV infection activated PKA activity as indicated by increased levels of phosphorylation of PKA substrates (Fig. 1D) (33). Also, here we showed that inhibition of PKA activity with inhibitor H89 in subgenomic HCV replicon cells repressed HCV replicon replication (Fig. 3B, C), suggesting that HCV replication is PKA-dependent. In addition, Michelle et al. reported the effects of PKA activity on HCV entry and infectivity (33). Inhibition of PKA activity induced a change of localization of receptor claudin-1, one of the host cell receptors required for HCV infection, from plasma membrane to intracellular vesicles leading to inhibition of HCV infection, suggesting that the plasma membrane localization of claudin-1 is PKA-dependent and is essential for viral receptor activity. We showed that targeting the ER stress/PKA/GSK-3β-dependent Wnt/β-catenin pathway with either PKA activity inhibitor H89 or ER stress inhibitor TUDCA both inhibited extracellular HCV infection (Fig. 5). Taken together, these results demonstrated that both PKA activity and ER stress is critical for HCV entry and replication.

## Conclusion

We demonstrated that Wnt/β-catenin signaling remained activated after HCV and replicon eradication by DAA in chronic HCV infection and HCV replicon. HCV infection activated PKA and PKA/GSK-3β-mediated Wnt/β-catenin signaling. PKA inhibition with H89 both repressed HCV and replicon replication and reversed PKA/GSK-3β-mediated Wnt/β-catenin signaling in chronic HCV infection and replicon. Both chronic HCV infection and replicon induced ER stress. ER stress inhibition with TUDCA repressed both HCV and replicon replication and reversed ER stress/PKA/GSK-3β-dependent Wnt/β-catenin signaling. Inhibition of either PKA or ER stress both inhibited extracellular HCV infection probably through inhibition of viral either entry or replication or both. Taken together, these results provide a potential novel therapeutic strategy for HCV-infected patients by targeting the ER stress/PKA/GSK-3β-dependent Wnt/β-catenin pathway not only to eradicate viruses but also reverse the Wnt/β-catenin pathway that is widely thought to be a major pathway in HCC, which overcomes the issue of DAA treatment that Wnt/β-catenin signaling remains activated despite HCV clearance.

## Supplementary Information

**Additional file 1.** Supplementary materials.

## Data Availability

Data will be available upon reasonable request from the corresponding author.

## Abbreviations

ATF6: Activating transcription factor 6
DAA: Direct-acting antiviral agents
ER: Endoplasmic reticulum
HCV: Chronic hepatitis C virus
PKA: Protein kinase A
TUDCA: Tauroursodeoxycholic acid
GSK-3β: Glycogen synthase kinase-3β
HCC: Hepatocellular carcinoma
UPR: Unfolded protein response
PERK: Protein kinase R-like endoplasmic reticulum kinase
IRE1: Inositol-requiring enzyme 1
