# Supplementary material for: Targeting ER stress/PKA/GSK-3β/β-catenin pathway as a potential novel strategy for hepatitis C virus-infected patients
Source: Cell Commun Signal. 2023 May 8;21:102. doi: 10.1186/s12964-023-01081-9 (PMC10165818; doi:10.1186/s12964-023-01081-9)
Supplement: Supplementary file 2 — Additional file 1. Supplementary materials. [file 12964_2023_1081_MOESM2_ESM.docx]

**Targeting ER stress/PKA/GSK-3β/β-catenin pathway as a potential novel strategy for hepatitis C virus -infected patients**

Dong Lin*1, Yijia Chen 2, Ali Riza Koksal 1, Srikanta Dash 1† and Yucel Aydin 1*†

1 Department of Pathology and Laboratory Medicine, Tulane University School of Medicine,

New Orleans, LA 70112, USA.

2 The college of liberal Arts and Sciences, Arizona State University, Tempe, AZ 85281, USA.

* Correspondence: dlin6@tulane.edu (D.L.); sdash@tulane.edu (S.D.); yaydin@tulane.edu (Y.A.)

† S.D. and Y.A. equally contributed to the study.


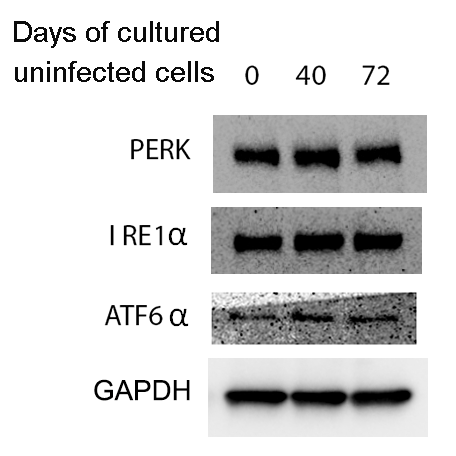


Figure S1. Whole cell lysates were taken from d40 and d72 of cultured uninfected Huh7.5 control cells for western blotting with indicated antibodies.


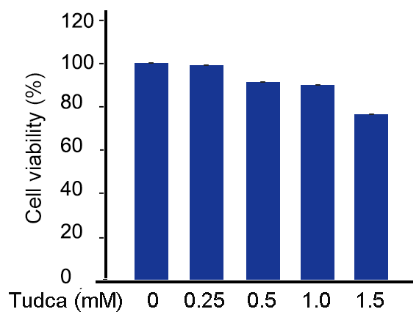


Figure S2. Cytotoxicity of TUDCA in chronic HCV-infected Huh7.5 cells (d54) was determined by MTT assay.
